# Investigation of sulphur isotope variation due to different processes applied during uranium ore concentrate production

**DOI:** 10.1007/s10967-016-4733-5

**Published:** 2016-02-22

**Authors:** Judit Krajkó, Zsolt Varga, Maria Wallenius, Klaus Mayer, Rudy Konings

**Affiliations:** 1 European Commission, Joint Research Centre (JRC), Institute for Transuranium Elements (ITU), Postfach 2340, 76125 Karlsruhe, Germany; 2 Faculty of Applied Sciences, Delft University of Technology, Mekelweg 15, 2629 JB Delft, The Netherlands

**Keywords:** Sulphur isotope, Uranium leaching, Uranium ore concentrate, Nuclear forensics, Process-related signature, Origin assessment

## Abstract

The applicability and limitations of sulphur isotope ratio as a nuclear forensic signature have been studied. The typically applied leaching methods in uranium mining processes were simulated for five uranium ore samples and the *n*(^34^S)/*n*(^32^S) ratios were measured. The sulphur isotope ratio variation during uranium ore concentrate (UOC) production was also followed using two real-life sample sets obtained from industrial UOC production facilities. Once the major source of sulphur is revealed, its appropriate application for origin assessment can be established. Our results confirm the previous assumption that process reagents have a significant effect on the *n*(^34^S)/*n*(^32^S) ratio, thus the sulphur isotope ratio is in most cases a process-related signature.

**Electronic supplementary material:**

The online version of this article (doi:10.1007/s10967-016-4733-5) contains supplementary material, which is available to authorized users.

## Introduction

Several new nuclear forensic signatures have been developed during the last years [1, 2]; however their routine application for real life investigation often leads to inconclusive decision except few notable ones, such as rare earth elements (REE) and isotope ratios of the major elements. This might be due to the fact that the persistence of most of these signatures during UOC processing has not been demonstrated or their variation in the course of the process has not been well understood. A more thorough study, however, would require a comprehensive set of samples from different origins following each process step and would limit conclusion to known processes.

In our previous study a novel method has been developed for the measurement of the *n*(^34^S)/*n*(^32^S) ratio in uranium ore concentrate (yellow cake) samples [3]. The sulphate content of UOC samples was leached with UP water and then pre-concentrated by anion exchange separation. Afterwards ^34^S/^32^S ratio was measured by multi-collector inductively coupled plasma mass spectrometry (MC-ICP-MS). By the application of the method for real UOC samples from different origins, the usefulness of sulphur isotope ratio as a nuclear forensic signature was investigated. Variations in sulphur isotope ratio is generally expressed as δ^34^S, the amount ratio of *n*(^34^S)/*n*(^32^S) of the sample relative to the IAEA V-CDT (Vienna Canyon Diablo Troilite) standard, expressed in ‰ and calculated using the following equation:

1 $$ \delta^{34}S_{\text{\{V-CDT\}}} (\permil) = \left[{\frac{{\left({\frac{{{}^{34}S}}{{{}^{32}S}}} \right)_{\text{sample}}}}{{\left({\frac{{{}^{34}S}}{{{}^{32}S}}} \right)_{{\text{V-CDT}}}}} - 1} \right] \times 1000 $$

where (^34^*S*/^32^*S*)_sample_ and (^34^*S*/^32^*S*)_V-CDT_ are the *n*(^34^S)/*n*(^32^S) of ratio of the sample and IAEA V-CDT standard, respectively. The (^34^*S/*^32^*S*)_V-CDT_ is defined as 0.0441626 ± 0.0000078 (*k* = 2) [4–6].

Our previous findings showed that δ^34^S value combined with ${\text{SO}}_{4}^{2 - }$ concentration can be a useful signature only for UOC samples originating from those sandstone type uranium deposits, where the uranium is leached with alkaline lixiviant (typically by in situ leaching), and not with sulphuric acid. Due to this process both their sulphate concentration and δ^34^S value are significantly lower, appearing as an individual group well separated from other UOC samples [3]. It was also observed that the majority of the investigated UOC samples have a δ^34^S value in the range of −5 to +15 ‰, which is consistent with the typical range of commonly used H_2_SO_4_ reagent [6]. It was also showed that in several cases the δ^34^S value differs from the sulphuric acid value, which suggests that the uranium ore can also contribute to the sulphur content of the final product. Nevertheless, for the majority of the samples, they cannot be distinguished from each other exclusively based on the difference in sulfur isotope ratio.

Comparison between results of UOC samples and literature values of corresponding ore deposits offers the possibility to identify potential correlations. In these considerations, however, a number of factors need to be taken into account. Several previous studies have been performed on the measurement of sulphur isotopic variation related to uranium deposits in order to reveal ore forming processes. However, they focused on the analysis of sulphur minerals (e.g.: pyrite, galena, sphaleryte) associated with uranium minerals or originating from the mineralised zone of the deposit, which may be different than the chemically processed UOC samples. Further complexity arises from the fact that such deposits may show largely varying sulphur isotope ratio throughout the ore body. This is due to biological and inorganic reactions involving chemical transformation of sulphur compounds leading to variations between −40 and +50 ‰ in different deposit types [7]. Several studies have been performed to find systematic changes in sulphur isotopic variation of different U-deposit [7–12], however the following overview will concentrate only on those deposit types where the samples used in this study originate from.

## Theory

Most of the publications [13–24] on sulphur isotope ratio variation in uranium deposits are related to* sandstone-type deposits*, in which pyrite plays an essential role in the uranium mineralization process. Sandstone-type deposits and in particular the roll-front subtype in Nebraska and Wyoming has been extensively studied by A. Meek [17]. Comparison with our study may be possible as her analysed samples were taken along the roll-front of the Three Crow deposit (7 km away from Crow Butte deposit—involved in present study), and represent the basal sands of the Lower Chadron Member, which hosts both uranium deposits. Very fine-grained pyrite, that is spatially associated with fine-grained coffinite crystals, has a wide range of δ^34^S values, from −43 to −16 ‰ and it is consistent with biological reduction or biologically induced chemical reduction. These are essential to the formation of this type of U deposits as e.g.: biogenically precipitated aqueous sulphides and pyrite transformed from iron oxides serve as the principal reductant of U(VI) to U(IV) in the Three Crow roll-front. In general, this range of δ^34^S value seems to be characteristic to the roll front type U-deposits [22, 25]. Northrop et al. [23] measured the δ^34^S values of sulphides from the Henry Basin, Utah, and showed that pyrite associated with mineralized samples has an average δ^34^S_sulphide_ value of −39.6 ‰, whereas Warren [20] measured an average −33 ‰ δ^34^S value of pyrite from the ore zone of Shirley Basin deposit, Wyoming.

Fewer studies can be found on samples originating from* unconformity type uranium deposits*. The majority of these publications are related to the Pine Creek Geosyncline in Australia and only some to the Athabasca basin, Canada. The geochemistry of Australian Pine Creek Geosyncline has been widely studied in the 1980’s. Unconformity type uranium deposits of South Alligator uranium district have been investigated by several research groups. Ayres and Eadington [25] measured sulphur isotopic variation in the Rockhole and El Sherana mine; δ^34^S values of minerals associated with pitchblende ores spread from −5.9 to + 12.3 ‰. Donelly and Ferguson [26] measured sulphur isotopic variation in samples originating from three uranium deposits, Jabiluka I-II-, Kongarra- and Ranger I. They found that sulphide samples present in ore zones have a range of δ^34^S values from −6 to +7 ‰, indicative of low-temperature biological sulphate reduction processes.

Alexandre et al. [27] analysed stable isotope variations (e.g.: N, C, S) in uraniferous bitumen originating from a sediment hosted unconformity type deposit in Southwest Athabasca. The measured δ^34^S varies from −4.2 to −2.7 ‰. Kotzer and Kyser [28] measured various sulphides and sulphates associated with U minerals from the Athabasca Basin. Isotopic results suggest mixing of basement fluid (δ^34^S values near 0) and basin fluid (near +15  ‰) during uranium mineralisation. However, late sulphides, developed during re-activation and incursion of low-temperature meteoritic waters, has highly variable δ^34^S values ranging from −25 to −5 ‰ and +15 to +40 ‰. In particular, J. Emberley et al. [29] investigated the petrography and chemistry of pyrite from the McArthur River uranium deposit. These samples were classified into six categories according to geological occurrence; in particular “ore-hosted pyrite” represents pyrite grains associated with uraninites. There is a large variation in S-isotopic compositions for pyrite within the deposit, δ^34^S varying from −30 to +40  ‰, but the values for pyrite associated with the U mineralization exhibit a fairly narrow, restricted range of δ^34^S values from 0 to +15 ‰, regardless of its occurrence. For ore-hosted pyrite this value was found to be −3 to +7 ‰.

Available sulphur isotope results related to *quart-pebble conglomerate deposits* are much more limited compared to sandstone and unconformity type deposits. Pyrite crystals of various size and morphologies from Stanleigh mine (Canada) shows wide range between −9.0 to +5.5 ‰ [30]. Watanabe et al. investigated samples from the Kaapvaal Craton (South Africa) and found that bulk-rock sulphides (mostly pyrite) range from +2.7 to +7.4 ‰ [31]. Isotope analyses of rounded pyrite grains from conglomerates of southern Africa (Zimbabwe, South Africa) indicate typically a small range of δ^34^S values close to that of igneous rocks (0 ± 5 ‰) with outliers having more positive values up to 16 ‰ [32].

To reveal further correlations between the δ^34^S value of the UOC and the uranium ore (or the respective deposit type), the major source of sulphur during the different uranium production steps should be understood. First, uranium ore is extracted from the deposit by traditional excavation (underground or open pit) or by alternative extraction method like “in situ leaching” (ISL).

The subsequent leaching of uranium from the ore can be either acid or alkaline depending on the gangue constituents. For acid leaching typically H_2_SO_4_ (10–100 kg t^−1^ ore) is used in the presence of an oxidant such as manganese dioxide or sodium chlorate to enhance solubility. Uranium is recovered from the leachate by ion-exchange (IX), solvent-extraction (SX) or direct precipitation. Uranium is obtained by eluting or stripping with an inorganic salt solution, such as sodium chloride or ammonium sulphate. When the carbonate content of ore makes acid leaching uneconomic, alkaline leaching is performed with sodium carbonate and bicarbonate solution. Uranium is recovered from the pregnant solution e.g. by sodium hydroxide precipitation [33, 34]. For ISL, both carbonate and acid leaching (dilute H_2_SO_4_) can be used depending on chemical and physical characteristic (e.g. permeability) of the ore horizon. Most frequently hydrogen-peroxide and oxygen are applied as oxidants, and uranium is recovered from the leach solution by ion exchange [35]. Subsequently the precipitate is filtered, dried and packaged for further processing.

Sulphate is introduced into the uranium hydrometallurgical process during the acid leaching (as H_2_SO_4_), elution of ion exchange or during back extraction following solvent extraction. Therefore it can be assumed that significant alteration both in the δ^34^S value and sulphate concentration in uranium ore concentrate samples arise from these steps.

In order to evaluate the applicability and limitations of sulphur isotope ratio as a nuclear forensic signature, we decided to carry out an investigation involving five uranium ore samples, whose corresponding UOC samples had been analyzed in our previous study. Different leaching methods typically applied in uranium mining industry were simulated for these five ore samples in order to (a) investigate the major source of the sulphur in the UOC samples, (b) to clarify whether the isotope ratio is indicative of the process and/or of the geological origin. The *n*(^34^S)/*n*(^32^S) ratio of the sulphuric acid used for the leaching was also measured in order to later estimate its contribution to the results. In addition, the sulphur isotope ratio variation was followed through two industrial sample sets from actual UOC production, in order to assess and compare the simulation results with real world samples.

## Experimental

### Instrumentation

A NuPlasma™ (NU Instruments, Oxford, United Kingdom) double-focusing multi-collector inductively coupled plasma mass spectrometer (MC-ICP-MS), equipped with 11 Faraday collectors and 3 discrete dynode electrode multipliers was used for the sulphur isotope ratio measurements. The instrument was operated at low mass resolution mode (*R* = 300). The samples were introduced into the plasma using a low-flow Teflon microconcentric nebulizer operated in a self-aspirating mode in combination with a desolvation unit (DSN-100, NU Instruments, Oxford, United Kingdom).

The sulphate measurements were performed by ion chromatography (IC). The ion chromatograph (Advanced Compact IC 861, Metrohm, Switzerland) is equipped with a chemical suppressor (Module MSM II) and a conductivity detector. The separation of sulphate was carried out using an anion exchange column (METROSEP A supp 5, 150 × 4.0 mm I.D.) preceded by a guard column (METROSEP Anion Dual 1, 50 × 4.6 mm I.D.).

Operating parameters of the ion chromatograph and optimized MC-ICP-MS instrumental settings with data acquisition parameters are given elsewhere [3].

### Reagents and materials

Ultra-pure water (UHQ System, USF Elga, Germany) was used for dilutions. Suprapur grade nitric acid (Merck, Darmstadt, Germany) was further purified by subboiling distillation and used for the sample preparation. All other reagents were of analytical grade. To prevent anionic contamination during the measurement, all lab ware was washed three times with ultra-pure water, dried in a laminar flow bench and stored in clean zipped bags. New and pre-cleaned labware was used for each sample.

The applied method was validated in previous paper [3]; however, silver sulphide reference materials (S-1, S-2, S-3), which are certified for sulphur isotope ratio and obtained from the International Atomic Energy Agency (IAEA) [5], were used as bracketing standards for sulphur isotope ratio measurement by MC-ICP-MS. For the analysis approximately 80 mg of each of the IAEA standards were weighed into a screw-cap Teflon vial and dissolved in 5 mL of nitric acid while heating to 95 °C on a hotplate for six hours. After cooling to room temperature, sulphate concentrations in these stock solutions were measured by IC. The stock solutions were subsequently diluted to 2 µg mL^−1^ (expressed as sulphur) in 1 % HNO_3_ for the sulphur isotope ratio measurement.

Five uranium ore samples (Table 1) originating from different mines were included in this study in order to investigate the variation of sulphur isotope ratio when applying different leaching methods. All samples were finely ground and carefully homogenized. For leaching suprapur grade sulphuric acid (Merck, Darmstadt, Germany) was used.

**Table 1:** Description of the investigated samples

Mine	Deposit type	Subtype	Country	Mining
McArthur River (McA)	Proterozoic unconformity	Basement-hosted	Canada	Underground
Rabbit Lake (RL)	Proterozoic unconformity	Basement-hosted	Canada	Open pit
Ranger (R)	Proterozoic unconformity	Basement-hosted	Australia	Open pit
Crow Butte (CB)	Sandstone	Rollfront	USA	ISL
SA Nufcor (SA)	Quartz-pebble conglomerate (QPC)	Quartzitic gold ore	South Africa	Underground

### Ore leaching methods and separation of sulphate

For the analysis of uranium ore concentrate samples, aqueous leaching was found sufficient to recover sulphur almost quantitatively [3]. In uranium ore samples, however sulphur can be present both as water leachable sulphate and non-soluble sulphur compounds. To account for this, three different leaching methods (Method I, II and III) were used to investigate the sulphur isotopic composition variation introduced by the process.

*Method (I)* approximately 200 mg of sample was taken and 10 mL ultra-pure water was added to it in a pre-cleaned plastic bottle.

*Method (II)* approximately 200 mg of sample was taken and 10 mL ultra-pure 0.01 M HNO_3_ was added to it in a pre-cleaned plastic bottle.

*Method (III)* approximately 300–500 mg of sample was weighed into a Teflon vial and leached in 7 mL 8 M ultra-pure nitric acid while heating to 90 °C on a hot-plate for 24 h. Approximately 200 µL of supernatant was weighed into a teflon vial and evaporated to dryness. Afterwards the residue was dissolved in 3 mL of ultra-pure water.

In order to measure the effect of chemical leaching on the original sulphur isotope ratio, industrial leaching methods were simulated (referred to later as Method IV) based on the real industrial conditions (Table 2).

**Table 2:** Conditions for the simulated industrial leaching (Method IV). δ^34^S values of corresponding UOC samples were obtained from [3] using Method I

Ore	Leaching	Oxidant	T (C°)	δ^34^S UOC [3]
McArthur River [36]	5 % H_2_SO_4_	O_2_	Ambient then 60	8.6 ± 1.1
Rabbit Lake [37] [38]	5 % H_2_SO_4_	NaClO_3_	65–75	14.9 ± 2.0
Ranger [39]	5 % H_2_SO_4_	MnO_2_	Ambient	7.25 ± 0.35
Crow Butte [40]	0.001 % NaHCO_3_	O_2_	Ambient	−12.3 ± 2.1
Nufcor	10 % H_2_SO_4_	MnO_2_	50–60	n.d.

All the samples were leached for 24 h at room temperature, centrifuged if necessary, and filtered with pre-rinsed 0.45 µm surfactant free cellulose acetate (SFCA) syringe filters (Nalgene, USA) before the ion-exchange separation. For the separation of ${\text{SO}}_{4}^{2 - }$ from the leaching solution anion exchange resin (AG 1-X4, Bio-Rad Laboratories, USA) was used. A complete description of the applied anion exchange separation can be found elsewhere [3]. Three replicates were measured for each sample.

### Measurement of ${\text{SO}}_{4}^{2 - }$ concentration and n(^34^S)/n(^32^S) ratio

Aliquots of 100 µl of the filtered leachate solutions were diluted to 10 mL with ultra-pure water. Approximately 0.5 mL was injected in the ion chromatography for the determination of ${\text{SO}}_{4}^{2 - }$ concentration in the samples. The relative combined uncertainty (*k* = 2) of the sulphate concentration by ion chromatography was less than 10 %.

Metal ions were removed from the sample solution by ion exchange separation prior to the mass spectrometric measurement in order to avoid isobaric interferences caused by doubly charged metals ions (e.g. ^64^Ni^2+^, ^64^Zn^2+^ or ^68^Zn^2+^). The use of the desolvating nebuliser system minimized the formation of oxide and hydrate species in the ICP-MS.

Sulphur isotope ratio was measured by MC-ICP-MS with blank1–standard–blank2–sample bracketing procedure, whereas sulphur concentration of the standards and samples for the MC-ICP-MS measurement was adjusted to approximately 2 mg mL^−1^ by dilution with 1 % HNO_3._ Silver (Ag) ICP standard solution, purchased from Alfa Aesar (Specpure^®^, Karlsruhe, Germany), served as (a) matrix matching for the bracketing standard as well as (b) avoiding the loss of sulphur via the applied desolvation system coupled to the MC-ICP-MS. It was added to the samples to obtain a final 4:1 molar ratio of Ag^+^/SO_4_^2−^.

All the other uncertainties are reported as expanded uncertainties (*U*) with a coverage factor *k* = 2. Uncertainty contributions from the measured *n*(^34^S)/*n*(^32^S) isotope ratios of the bracketing standard (IAEA-S-1) and the sample, the isotope abundance ratio of the IAEA-S-1 (0.0441493 ± 0.0000080, *k* = 2), and the uncertainty of the assigned V-CDT δ^34^S value (0.0441626 ± 0.0000078, *k* = 2) [5] has been taken into account to calculate measurement uncertainty.

## Results and discussion

### The variation of n(^34^S)/n(^32^S) ratio in uranium ores

**Fig. 1:**
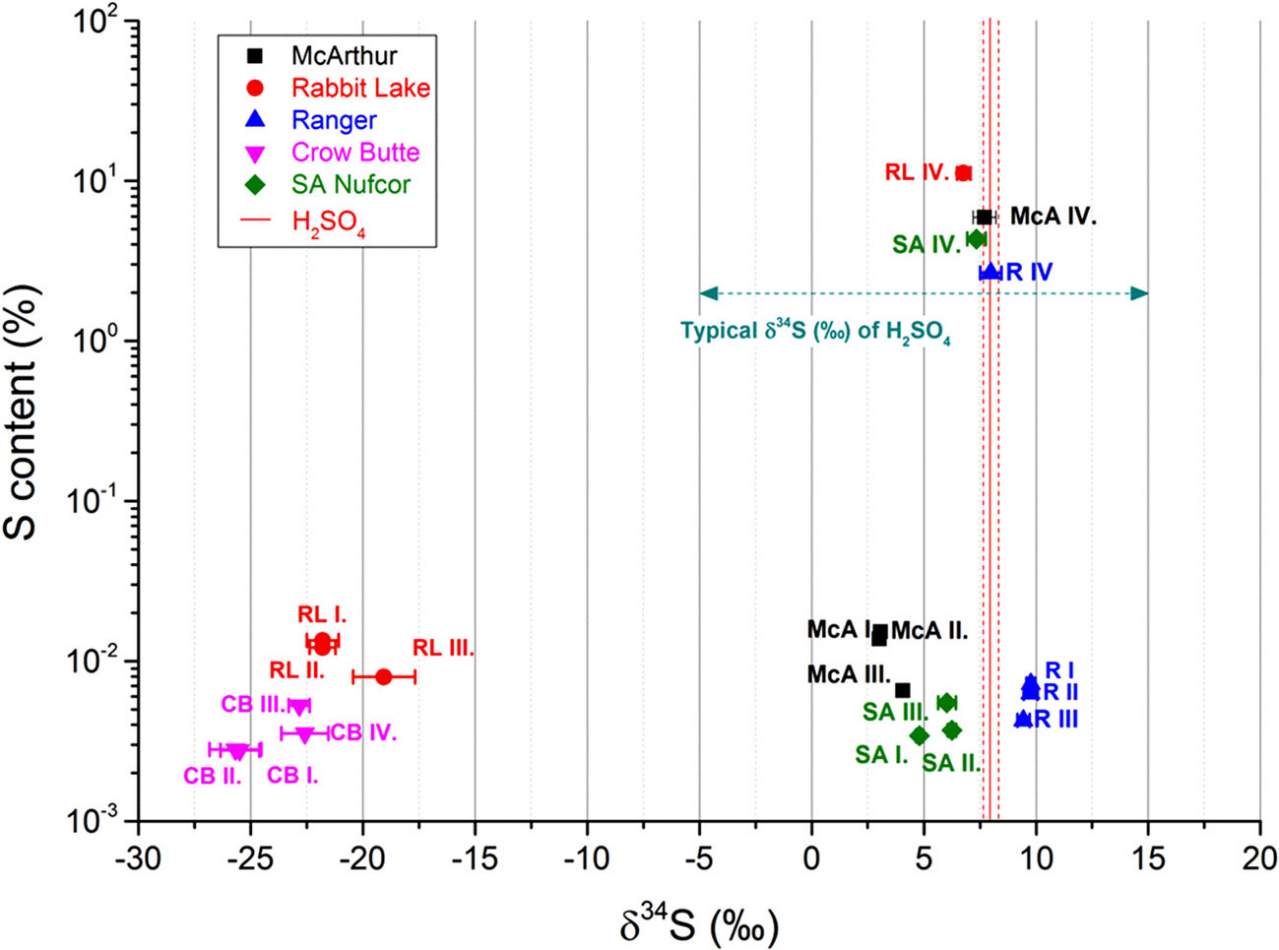
Distribution of δ^34^S (‰) and sulphate concentration of the analysed uranium ore leachate samples. δ^34^S (‰) value of sulphuric acid used for leaching in this study as well as typical δ^34^S (‰) value of commercial sulphuric acid are also shown

Sulphur isotope ratio and sulphate concentration results are summarised in Table 3 and depicted on Fig. 1. With regard to the different leaching methods, we can observe for all samples significant differences in the measured δ^34^S value between Method IV and the other three (Method I-III) when using H_2_SO_4_ leaching. It is apparent from Fig. 1 that the measured δ^34^S and sulphur concentration values of Method I-III are scattered close to each other. As it was expected samples from Method IV have δ^34^S values close to that of sulphuric acid (7.96 ± 0.19 ‰) used for leaching, which also explains the higher sulphur quantity. In case of Crow Butte sample there is a small, but observable difference in the isotope ratio between Method I-II and Method III-IV. As during the process of Crow Butte there is no sulphuric acid added to the sample, we can assume that variation of δ^34^S value is likely caused by the different solubility of various sulphur minerals.

**Table 3:** Measured δ^34^S results by the different leaching methods and the calculated process contribution of sulphuric acid

Ore samples	${\text{c}}_{{{\text{SO}}_{4}^{2 -}}}$ in leachate (µg/g)	δ^34^S (‰)	*α*_process_ (%)
McArthur I	138	3.01 ± 0.16	95 ± 12
McArthur II	153	3.07 ± 0.16	
McArthur III	65	4.06 ± 0.24	
McArthur IV	59,300	7.70 ± 0.51	
Rabbit Lake I	135	−21.8 ± 0.70	96 ± 4
Rabbit Lake II	122	−21.8 ± 0.57	
Rabbit Lake III	78	−19.1 ± 1.4	
Rabbit Lake IV	111,500	6.77 ± 0.30	
Ranger I	72	9.76 ± 0.20	99 ± 30
Ranger II	63	9.75 ± 0.30	
Ranger III	43	9.44 ± 0.27	
Ranger IV	26,500	6.93 ± 0.40	
Crow Butte I	28	−25.7 ± 1.2	
Crow Butte II	28	−25.5 ± 0.87	
Crow Butte III	53	−22.8 ± 0.47	
Crow Butte IV	35	−18.5 ± 7.0	
SA Nufcor I	34	4.80 ± 0.16	80 ± 15
SA Nufcor II	37	6.25 ± 0.19	
SA Nufcor III	55	6.03 ± 0.40	
SA Nufcor IV	43,300	7.01 ± 0.29

Further evaluation was carried out, in order to estimate quantitatively the alteration of the δ^34^S value by the process reagents. Previous research on application of Pb isotope ratio as nuclear forensic signature showed similar problem, namely that radiogenic lead in the U ore was first separated during purification steps and later diluted with natural lead originating as contaminant of the process. Varga et al. demonstrated however, that by calculating the contribution of natural lead to the sample, one can partly overcome this problem [41]. In analogy, we built a model where we assumed that original δ^34^S value of the ore deposit is close to the result obtained by leaching (Method I). For samples where H_2_SO_4_ was used for leaching (Method IV), the δ^34^S is expected, and as it was proven, to be different from the original value closer to the value of the used sulphuric acid.

The relative contribution of “process contamination” during sulphuric acid leaching for each sample (*α*_process_) has been estimated using the following equation (Eq. 2):

2 $$\alpha_{\text{process}} \; = \;\left({\frac{{\delta {}^{34}S_{I} - \delta {}^{34}S_{IV}}}{{\delta {}^{34}S_{I} - \delta {}^{34}S_{{H_{2} SO_{4}}}}}} \right)\; \times \;100$$

where *δ*^*34*^*S*_*I*_ and *δ*^*34*^*S*_*IV*_ are the measured δ^34^S values of samples leached with Method I and Method IV, while $\delta {}^{34}S_{{{\text{H}}_{ 2} {\text{SO}}_{ 4}}}$ is the average isotope ratio value (7.96 ± 0.19  ‰) (*n* = 4) of sulphuric acid applied as leaching reagent in our experiments. Results showed that contribution of sulphuric acid reagent on McArthur River, Rabbit Lake and Ranger samples is between 95–99 %, while in case of South African ore the process contribution is about 80 % (Table 3). Therefore, one can conclude that for samples where H_2_SO_4_ leaching is used, the determined δ^34^S value reflects largely, if not fully, the δ^34^S value of used sulphuric acid.

Measurement of McArthur River samples resulted in δ^34^S values between +3 and +4. This finding is in good agreement with the measured δ^34^S values for ore-hosted pyrite (−3 to +7 ‰) from McArthur River deposit [29]. Moreover, previously measured UOC samples [3] showed a value of 8.6 ± 1.1 ‰, hence in good agreement with the result of 7.7 ± 0.51 ‰ obtained by the Method IV (industrial leaching) (Table 2 and 3). This finding indicates that δ^34^S values of sulphur bearing process chemicals might be close to that we used for our simulations.

Our findings for the other unconformity type mines (Rabbit Lake and Ranger by Method I–III) are also in accordance with the previous studies confirming the relative large range of δ^34^S values from −25 to 40 ‰ found earlier [7, 26, 27]. The results on Crow Butte samples are also consistent with results of previous studies on roll-front type U deposits [17, 20, 23]. Moreover, it could be demonstrated that the industrial leaching (Method IV) does not largely affect the original δ^34^S value as no H_2_SO_4_ is applied.

Results of Nufcor samples show δ^34^S values between 4.8 and 6.25 ‰. Although δ^34^S value of H_2_SO_4_ is quite close to the values of Nufcor ore (Method I–III), it still has significant effect during the leaching shifting towards its δ^34^S value to 7.01 ‰ (Method IV). By comparing results with literature data we can conclude that they are consistent with general QPC trends for southern African samples, namely having more positive values between −5 to 16 ‰ [31, 32].

### Variation of n(^34^S)/n(^32^S) ratio in UOC production

Aqueous leaching (i.e., method I) was also applied for the measurement of intermediate products in the course of UOC production. The aim was to support our results of different leaching tests by the measurement of samples coming from industrial processes and facilities. The samples originate from Nufcor, South Africa and Olympic Dam, Australia. Below are summaries of the applied processes in both UOC production facilities, respectively.

UOC is produced in the Nufcor facility by the following process: Uranium ore is leached with sulphuric acid. Ion exchange (IX), followed by solvent extraction (SX) are used to purify the acidic leachate. For the elution (IX) and the back-extraction (SX) of the uranium 12 % sulphuric acid and ammonium sulphate is used, respectively. In the presence of ammonia, uranium is precipitated as ammonium di-uranate (ADU). The ADU slurry (15 wt% U_3_O_8_) is then filtered and dried to ADU powder, which is finally calcined to U_3_O_8_ at 490 °C. Samples were collected at each stage of the process in order to follow the flow of material originating from the same feed.

At Olympic Dam site, after crushing and grinding, the ore is subjected to a flotation circuit, where uranium containing ore is separated from tailings with approximately 90 % efficiency. The uranium is leached with sulphuric acid in the presence of NaClO_3_ oxidant at approximately 50 °C. After residual copper is separated, uranium is further purified by SX circuits. Stripping is done with ammonium sulphate and precipitated as ammonium di-uranate (ADU). The final oxide product (U_3_O_8_) is obtained by calcination of dried ADU at about 760 °C [39]. The investigated samples include uranium ore, ADU and calcined U_3_O_8_. Samples were collected during fall of 2001 and are assumed to represent consecutive production steps.

**Fig. 2:**
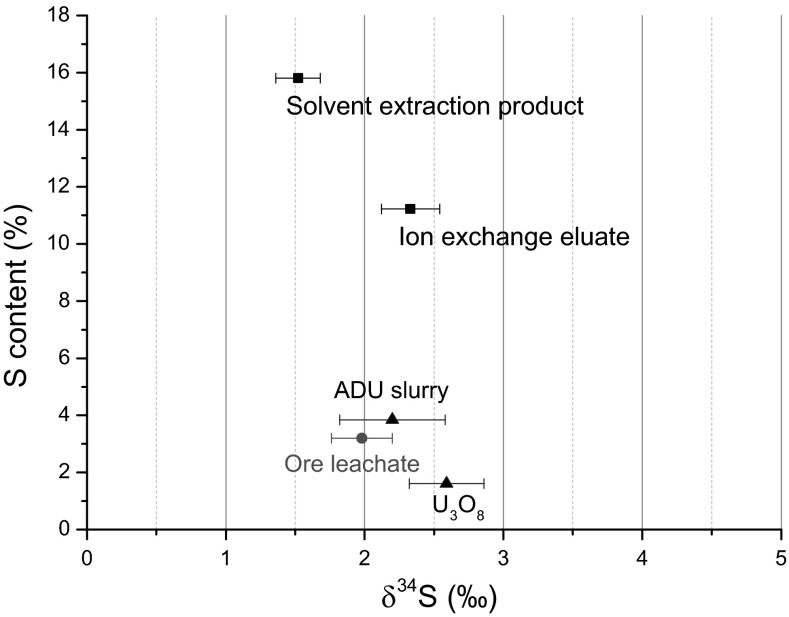
Distribution of δ^34^S (‰) and relative sulphate concentration of the Nufcor samples during the UOC production

**Fig. 3:**
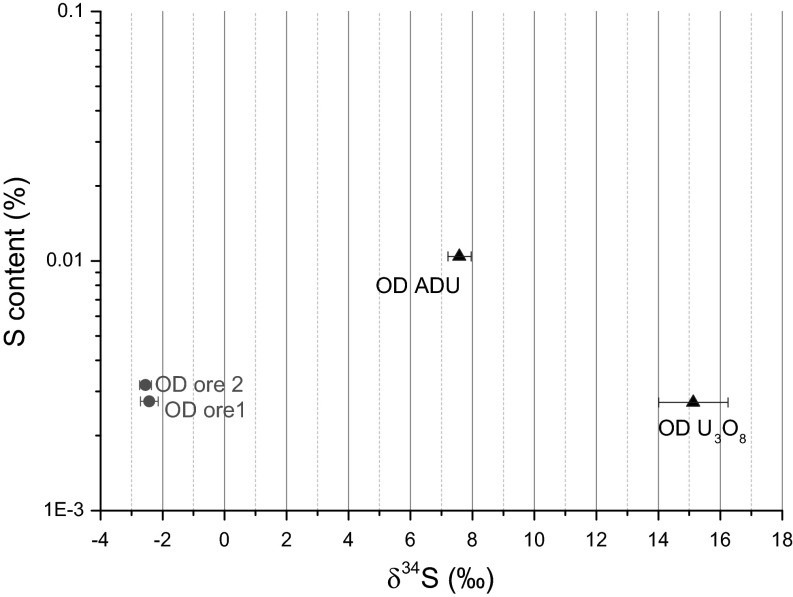
Distribution of δ^34^S (‰) and relative sulphate concentration of the Olympic Dam (OD) samples during the UOC production

The sulphur concentration and the *n*(^34^S)/*n*(^32^S) were measured on the respective samples using aqueous leaching (hence, Method I) and the results are shown in Figs. 2 and 3. As can be seen from Fig. 2, the sulphur concentration in the investigated Nufcor samples is significantly increasing from the ore to the samples representing solvent and ion extraction stages. This is obviously due to sulphur containing reagents added in large amounts during leaching, IX and SX circuits. During any of these steps *n*(^34^S)/*n*(^32^S) value does not change notably, whilst the sulphur concentration is later significantly reduced by the precipitation of ADU and by the calcination of ADU to the final oxide product (U_3_O_8_).

When we compare the simulated leaching results (Table 3) and the real industrial samples of Nufcor, we can see that the initial δ^34^S value of the ore measured by Method I-III is 5.69 ± 0.25 ‰. In the real samples we can see that the ore leachate is 1.98 ± 0.22 ‰, while IX and SX is 2.33 ± 0.21 ‰ and 1.52 ± 0.16 ‰, respectively. This result supports our finding that leaching is the step which significantly changes the initial ore value. Moreover, the results clearly show the change, in both the δ^34^S value and sulphate concentration, caused by sulphur bearing solvents during IX and SX.

The results for sample set originating from Olympic Dam are shown in Fig. 3. As expected, the sulphur contribution of process chemicals has an obvious effect on the sulphur isotope ratio in ADU and U_3_O_8_ products, resulting in a shift towards positive δ^34^S values. The sulphate concentration increases from the ore to ADU and decreases then again once the material is calcined from ADU to U_3_O_8_. Associated with the latter process step we observe also a shift towards higher δ^34^S values. This change might attributed to isotopic fractionation occurring during calcination (at high temperature) involving the preferential evaporation of the lighter (sulphur) isotope.

In summary the results of both real sample sets support the results obtained from our leaching studies where we demonstrated that the sulphur isotopic signature of the ore is altered due to the high amount of sulphur containing reagents added to the material flow during the UOC production process, thus reflecting at the end the δ^34^S value in the used sulphur containing reagents.

## Conclusion

The present study was undertaken to further evaluate the suitability of the sulphur isotope ratio as indicator of the origin or processing history of uranium ore concentrates, hence as a nuclear forensic signature. In particular, we investigated the impact of sulphur isotope alteration caused by process chemicals used for the production of uranium ore concentrates. The findings of this investigation complement those of our earlier studies [3] and the following conclusions can be drawn:

In case uranium leaching is performed in the absence of sulphuric acid (e.g. in in situ leaching where NaHCO_3_ is used as lixiviant), the sulphur isotope ratios measured in the ore concentrate samples reflect the values observed for the ore. Hence, in this case the sulphur isotope ratio provides an additional hint on the geological origin of the uranium. When sulphuric acid is used as leaching agent (or for back-extraction of uranium during purification), the sulphur isotope ratio will essentially reflect the values of the sulphur containing reagents used for processing the ore.

The findings of this study, based on a combination of different leaching tests and the investigation of the sulphur isotope ratio variation during UOC production from ore to U_3_O_8_ product in real industrial samples, showed that process reagents have a significant effect on the *n*(^34^S)/*n*(^32^S), thus the sulphur isotope ratio is largely a process-related signature.

## Electronic supplementary material

Below is the link to the electronic supplementary material.

Supplementary material 1 (PDF 100 kb)
