# Supplementary material for: Investigation of sulphur isotope variation due to different processes applied during uranium ore concentrate production
Source: J Radioanal Nucl Chem. 2016 Feb 22;309:1113–21. doi: 10.1007/s10967-016-4733-5 (PMC4990604; doi:10.1007/s10967-016-4733-5)
Supplement: Supplementary file 1 — Supplementary material 1 (PDF 100 kb) [file 10967_2016_4733_MOESM1_ESM.pdf]

Investigation of sulphur isotope variation due to different processes applied during uranium ore concentrate production

Judit Krajkó • Zsolt Varga • Maria Wallenius • Klaus Mayer • Rudy Konings

Supplementary information - Table 1. Summary of $\delta^{34}S$ (‰) values measured in different uranium deposits

Uranium deposit type	Mine	Geological formation	Country	$\delta^{34}S$ (‰)	Sample	Ref.
Methamorphite	Shinkolobwe	Zaire	D. R. of Congo	-1.3 to +6.9	pyrite	[9]
Collapse Breccia Pipe	Orphan Lode	Arizona	USA	-27.2 to -2.8	pyrite	[10]
Granit related	main uranium deposits in southern		China	+2.7 to +7.4	bulk-rock sulfides	[11]

	Zhuguang granitic batholith					
Vein	Xiazhuang	Xiazhuang ore field	China	-15.3 to -4.9	pyrite associated with uraninite	[12]
Sandstone	Beverley North	Lake Eyre Basin	Australia	-43.8 to +32.4	pyrite	[13]
				- 43.8 to - 18.3	framboid pyrite	
	Felder	South Texas	USA	<-20	pyrite formed before and	[14]
	Lamprecht				during U precipitation	
	Benavides			< -20	pyrite formed before and during U precip.	
	Panna Maria			-34 to -1	sulphides associated with uranium	
	at several districts of the Colorado			-5 to +26		[15]

Plateau uranium mining area					
	Texas	USA	-40 to -25	Pyrite, marcasite	[16]
Three Crow	Wyoming	USA	-32 to -16	pyrite	[17]
SM-18 and Jo Dandy	Colorado	USA	-13.3 to -5.0	pyrite that is spatially associated with U mineralization	
Tono		Japan	10 to 43	pyrite and gypsum	[18]
	Wyoming	USA	-20.5 to -17.8		[19]
Shirley Basin	Wyoming	USA	-33	pyrite from ore zone	[20]
Beverley	Lake Frome Basin	AUS	0.7 to 1.3	pyrite associated with coffinite	[21]
			− 35.5 to −26.2	authigenic sulfides	

	Henry Basin	Utah	USA	-48 to -22	sulphide	[23]

continued on next page

**Uranium deposit type**	**Mine**	**Geological formation**	**Country**	**δ^{34}S (‰)**	**Sample**	**Ref.**
Unconformity	El Sherana, Rockhole	South Alligator district	Australia	-1.2	bedded sulphides (dom. Pyrite)	[25]
				-6 to +12	vugs and vein sulphides	
	Jabiluka I-II., Kongarra, Ranger I.			-0.5 to +3.5	bedded sulphides (dom. Pyrite)	[26]
				-6 to +7	sulphides in vugs and veins within the brecciated U mineral zones	
	subeconomic U-mineralization	Sothwest Athabasca	CAN	–4.2 to –2.7	uraniferous bithumen	[27]
		Athabasca Basin	CAN	-25 to -5 and +15 to	sulphate minerals associated with U deposits	[28]

				+40‰.		
	McArthur River			0 to + 15	pyrite associated with the U mineralization	[29]
				-3 to +7	ore-hosted pyrite	
Quartz-pebble-conglomerate	Stanleigh	Elliot Lake, Matinenda Formation, Ontario	Canada	−9.0 to +5.5	pyrite crystals (73 analyses) of various size and morphologies	[30]
		Kaapvaal Craton	South Africa	2.7 to 7.4	bulk-rock sulfides (mostly pyrite)	[31]
		Dominion Reef		-1.0 to 7.6	Rounded massive pyrite	
				around 0	Intergrown chalcopyrite and pyrite from a quartz vein in the underlying basement granite	

			cubic pyrite in the overlying volcanic rocks
Mozaan Contact Reef	Pongola Basin.	0.2 to 2.9	Rounded, massive pyrites
Kimberley Reef	Witwatersrand Supergroup	-0.2 to 3.7	Rounded pyrites, both massive and porous,
Ventersdorp Contact Reef	Ventersdorp Supergroup,	-1.5 to 4.3	Rounded and cubic pyrites
Black Reef	Transvaal Basin	-5.6 to 3.2	Rounded pyrites
